# Clinical efficacy of a thermosensitive embolic agent for the treatment of pediatric renal vascular hypertension due to renal artery branch stenosis in children

**DOI:** 10.3389/fped.2025.1638141

**Published:** 2025-10-16

**Authors:** Yakun Wang, Xingmiao Liu, Ji Cheng, Dong Li, Yang Liu

**Affiliations:** ^1^ Pediatric Cardiovascular Center, Tianjin Children's Hospital/Tianjin University Children's Hospital, Tianjin, China; ^2^ Department of Pediatric Neurology, Tianjin Children's Hospital/Tianjin University Children's Hospital, Tianjin, China

**Keywords:** thermosensitive embolic agent, renal artery branch stenosis, renal vascular hypertension, pediatric patients, interventional embolization, headache relief, blood pressure, renin levels

## Abstract

**Objective:**

To evaluate the clinical efficacy of a thermosensitive embolic agent in the treatment of renal vascular hypertension caused by stenosis in the renal artery branches.

**Methods:**

We retrospectively analyzed 18 pediatric patients who were admitted to our hospital between March 2020 and January 2024 for interventional embolization due to hypertension caused by stenosis of renal artery branches. We compared the degree of headache relief, blood pressure, cure rate, improvement rate, changes in target organ damage, and renin levels before and after the treatment.

**Results:**

(1) Clinical symptoms significantly improved, with a marked relief of headache and significant reduction in NRS-11 scores (*p* < 0.05). (2) Blood pressure control showed obvious improvement, with both systolic and diastolic pressures significantly lower than pre-intervention levels (*p* < 0.05), and the use of antihypertensive medications were reduced (*p* < 0.05). (3) At the 3-month follow-up, the cure and improvement rates were 55.6% (10/18) and 44.4% (8/18), respectively. At 6–12 months, the cure rate increased to 77.8% (14/18), while the improvement rate showed a corresponding decrease to 22.2% (4/18). (4) Improvement in target organ damage: At the 3-month post-intervention follow-up, echocardiographic findings of all children had returned to normal, with no signs of left ventricular hypertrophy. Both serum creatinine(Scr) and blood urea nitrogen(BUN) levels also returned to normal. Additionally, the 24 h urinary protein quantification at 3 months post-intervention was significantly lower than pre-intervention levels. During the 6–12 month follow-up period, except for one child with a mild abnormality, urinary protein indicators of all other children remained at normal levels. (5) Changes in renin levels: during the intervention, the affected renal vein demonstrated significantly elevated renin levels compared to both the contralateral renal vein and inferior vena cava (*p* < 0.05), while post-intervention peripheral venous renin levels were significantly lower than pre-intervention values (*p* < 0.05).

**Conclusion:**

Interventional therapy using thermosensitive embolic agents for pediatric renovascular hypertension caused by renal artery branch stenosis demonstrated significant clinical efficacy. It effectively improved the clinical symptoms, blood pressure control, target organ damage, and renin levels of the children, with no serious complications observed during follow-up. This provides a new option for clinical treatment.

**Level of Evidence:** Level 4, an uncontrolled clinical intervention study.

## 1 Introduction

The incidence of hypertension is increasing in the modern society, making it one of the most common chronic diseases. According to a sampling survey conducted from 2012 to 2015, the crude prevalence rate of hypertension among urban and rural residents aged 18 years and above in China reached 27.9% (1). As a chronic disease with various complications, hypertension affects not only middle-aged and elderly people but also children. In 2019, Xi et al. found that the incidence of three-time-point hypertension among children aged 12–17 years in China was as high as 5.9% (2).

Renal vascular hypertension (RVH) reportedly accounts for 5%–25% of pediatric hypertension cases and is primarily caused by renal artery stenosis (RAS) and mid-aortic syndrome (MAS). Among these cases, approximately 50% involve stenosis of the secondary renal artery branches, while stenosis of the main trunk and distal branches account for 25% and 12.5%, respectively (3). However, therapeutic options for branch renal artery stenosis are limited. While endovascular angioplasty is more suitable for larger vessels, is not applicable to small renal artery branches located within the renal parenchyma. Surgical wedge resection is associated with significant trauma. As a result, Branch renal artery embolization has emerged as the primary treatment option for this condition (4, 5). This article reports on 18 pediatric patients with renovascular hypertension caused by renal artery branch stenosis who underwent endovascular treatment using a thermosensitive embolic agent. The aim of this study is to analyze the clinical efficacy of this interventional procedure.

## 2 Materials and methods

### 2.1 Patient selection

A total of 18 children aged 6–15 years (mean age: 9.7 ± 2.3 year) with renal artery branch stenosis who underwent interventional embolization at Tianjin Children's Hospital between March 2020 and January 2024 were included. Among these participants, 10 and 8 were boys (55.6%) and girls (44.4%), respectively.

The inclusion criteria were as follows: (1) All patients were prescreened by hypertension specialists and nephrologists. Children with resistant hypertension were selected, defined as those whose blood pressure remained above the level of grade 2 hypertension according to the “Chinese Reference Standards for Blood Pressure by Gender, Age, and Height in Children Aged 3–17 years” (6) despite adherence to a regimen of at least one antihypertensive medication (calcium channel blocker) for a minimum of four weeks. Blood pressure measurements were obtained by averaging office readings taken on three different days. (2) Renal Computed Tomography Angiography (CTA)/Digital subtraction angiography (DSA) demonstrating significant stenosis of a single tertiary and lower-order branch of the renal artery, interlobar artery stenosis distal to the segmental arteries, presence of wedge-shaped perfusion defects, or children in whom renal artery angioplasty could not be performed. (3) Normal preoperative renal function or Scr ≤ 2 times the baseline value.

The exclusion criteria were as follows: (1) Hypertension caused by other renal parenchymal, cardiac, or endocrine-related diseases. (2) Significant coagulation abnormalities or other contraindications for intervention. (3) Contraindications to antiplatelet drugs.

Children who met the inclusion criteria were referred to the vascular interventional center for renal artery embolization with thermosensitive embolic agents.

### 2.2 Renal CTA

**Figure 1:**
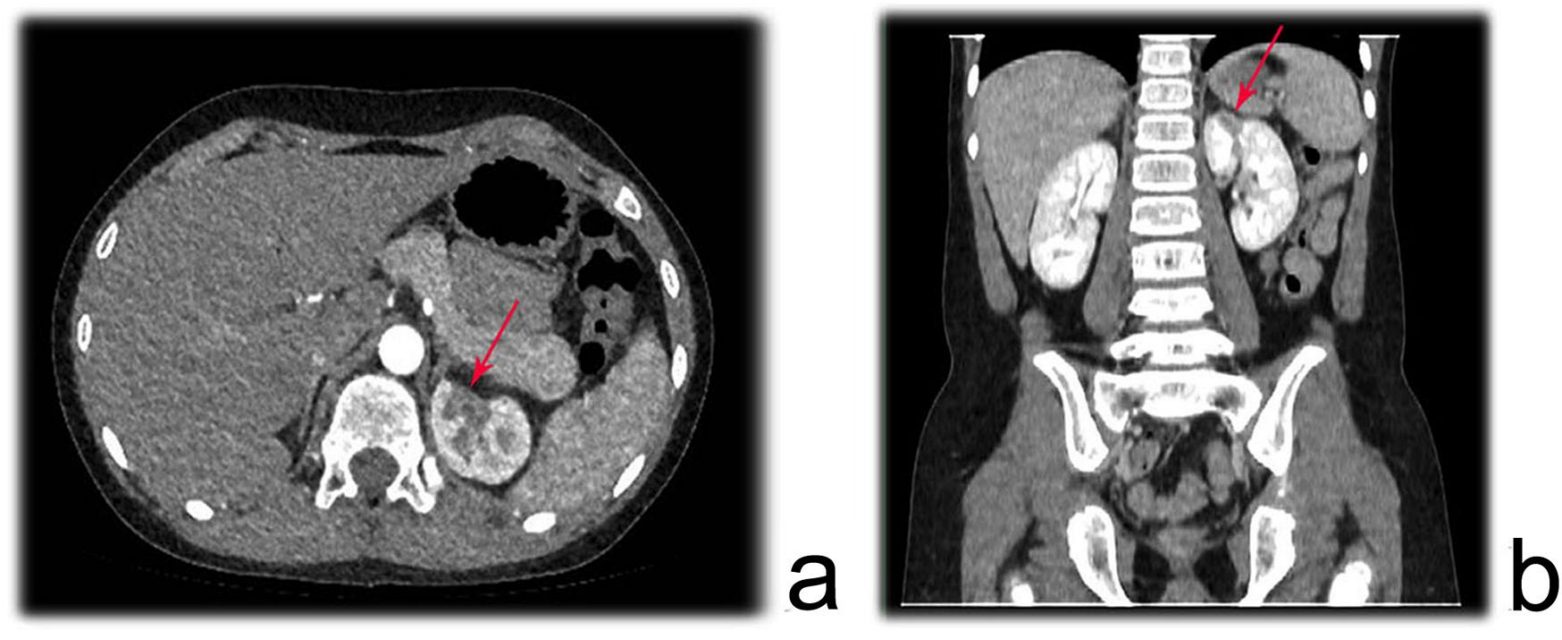
Renal CTA showing a region of reduced perfusion in the upper pole of the left kidney. **(a)** axial plane; **(b)** Coronal plane.

Prior to the examination, the child was required to fast for 4–6 h to reduce the likelihood of adverse reactions to the contrast agent. For children who were uncooperative, oral administration of 10% chloral hydrate at a dose of 0.4–0.5 ml/kg was required for sedation. A non-ionic iodinated contrast agent with a concentration of 300 mgI/ml was administered via a 22- to 24-gauge peripheral intravenous catheter placed in the right upper limb or both lower limbs, with an injection rate of 0.07 ml kg^−^¹ s^−^¹ (22-gauge <3.0 ml/s, 24-gauge <1.5 ml/s). All children underwent spiral volumetric scanning of the renal artery phase using a CT scanner with more than 128 detector rows, initiated 22–24 s after the start of contrast injection. After scanning, a set of axial images with a resolution of 0.625 × 0.4 mm was used for three-dimensional vascular volume rendering (VR) reconstruction to evaluate the morphology, structure, course, and degree of stenosis of the renal artery. Figure 1 illustrates a region of reduced perfusion in the upper pole of the left kidney on renal CTA.

### 2.3 Renal DSA

Digital subtraction angiography (DSA), with its superior spatiotemporal resolution, remains the gold standard for diagnosing renal artery stenosis, demonstrating unique advantages in detecting stenoses in secondary and higher-order branches (7). Prior to the procedure, informed consent was obtained, followed by a 6-hour fasting and bowel preparation. Under general anesthesia, the right femoral artery was punctured under ultrasound guidance, and a pigtail catheter was introduced to perform non-selective renal arteriography. Initial angiographic images provided clear visualization of the bilateral main renal arteries. Subsequently, a 4F C2 catheter was selectively advanced into the left and right main renal arteries, and a contrast medium was injected for dynamic DSA acquisition. This technique enabled precise identification of stenoses in the main renal arteries and their branches, including the degree of narrowing and distal perfusion status.

### 2.4 Thermosensitive embolic agent

This study utilized a commercially available temperature-sensitive liquid embolic agent, Pepuxin (Beijing Guanhe Medical Technology Co., Ltd., National Medical Device Approval No. 20203130345). The primary component of this thermosensitive embolic agent is a copolymer of N-isopropylacrylamide and N-propylacrylamide (crosslinked with N, N-methylenebisacrylamide) combined with the radiopaque agent iohexol and a saline solvent. The embolic agent exhibits temperature-dependent physical properties, with a phase transition temperature range of 30–35°C. Below this threshold temperature, the agent remains in liquid form. Once it reaches body temperature (above the phase transition temperature), the polymer forms and gradually precipitates from the solvent, thereby achieving vascular embolization. According to product specifications, the agent was injected slowly through a microcatheter under DSA guidance at an injection rate of 1.5–3 mL/min. During administration, the catheter was cooled with 1% heparinized saline to prevent premature phase transition within the catheter and ensure procedural safety.

### 2.5 Renal artery embolization using a thermosensitive embolic agent

**Figure 2:**
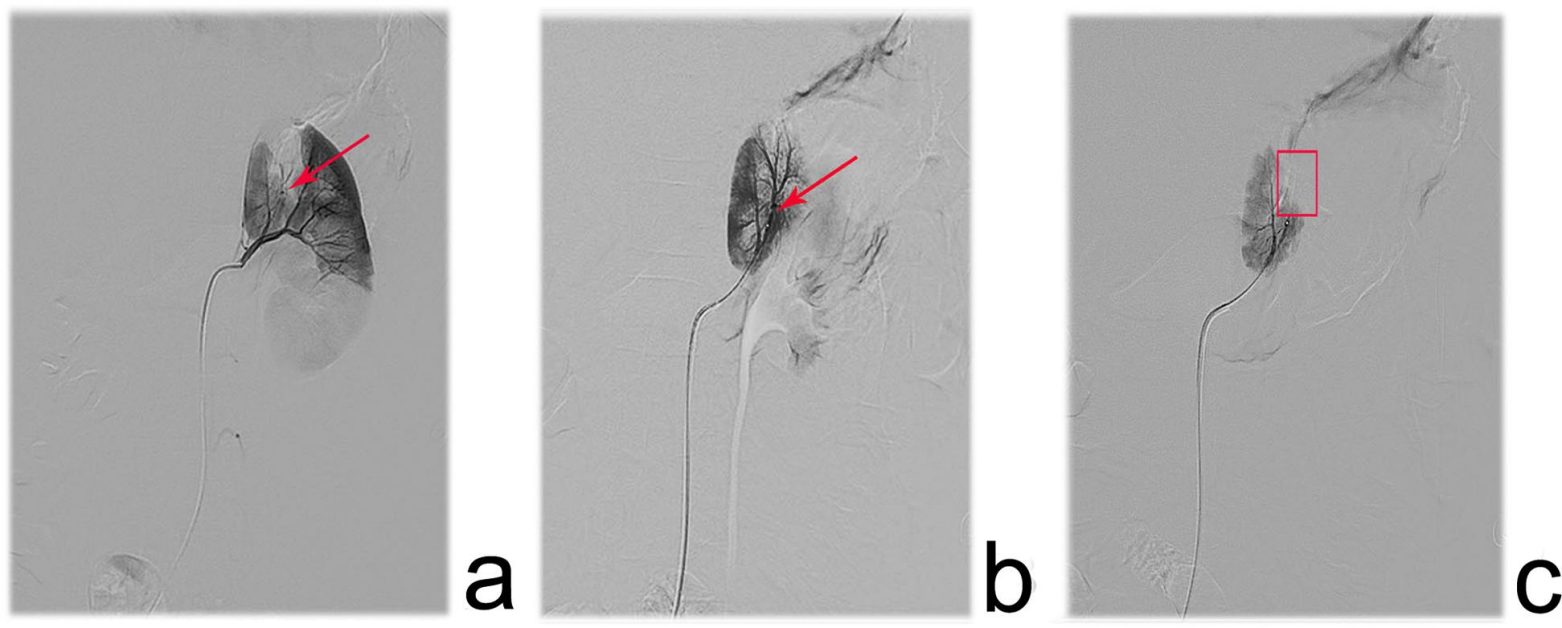
Angiographic images depicting the surgical procedure in pediatric patients with renal artery branch stenosis. **(a)** one interlobar artery in the upper segment of the left renal artery showed linear stenosis(red arrow), and the renal parenchyma distal to the stenosis exhibited poor opacification. **(b)** a 1.98 F microcatheter was advanced over a 0.018‘’ micro guidewire to the proximal end of the stenosis, and branch stenosis was obvious. **(c)** The post-intervention angiography performed complete occlusion of the stenotic vessel, with no residual blood flow and loss of opacification in the affected renal region.

After administering anesthesia, the patient was positioned supine and both groin areas were routinely sterilized. Using the Seldinger technique, the right femoral artery and vein were successfully punctured, and 4F vascular sheaths were inserted. A C2 glide catheter was selectively advanced into the left and right renal veins, as well as the inferior vena cava distal to the renal vein ostia, with 2 mL blood samples collected from each site for renin, BUN, and Cr analysis. A 4F pigtail catheter was then introduced via the right femoral sheath and positioned at the T12 level for anteroposterior angiography to assess renal vascular anatomy and perfusion. Subsequently, selective angiography of the affected renal artery was performed using a glide-assisted single-curve catheter via the guiding catheter, enabling a detailed assessment of interlobar and interlobular stenosis (including severity and length) and distal renal perfusion. If angioplasty was not suitable, embolization was performed based on the findings. The microcatheter/microguidewire system was navigated to the proximal segment of the stenotic vessel. Under fluoroscopic guidance, Pepuxin was slowly injected at 1.5–3 mL/min until flow decelerated or stasis occurred. Care was taken to prevent reflux into the normal vasculature and minimize the risk of complication. Prior to completing embolization, a heparinized saline flush cooled the catheter to avoid an intraluminal phase transition. Post-embolization angiography confirmed therapeutic efficacy, and repeat embolization was performed if suboptimal occlusion was observed until complete flow cessation and territorial non-opacification were achieved. Finally, the catheters and sheaths were removed with manual compression, and a pressure dressing was applied to ensure hemostasis. Figure 2 depicts the endovascular intervention for a stenotic interlobar artery, progressing from initial diagnosis and catheterization to successful vessel occlusion.

### 2.6 Data analysis

This study collected and analyzed patient demographics, clinical characteristics, pre-intervention assessments, laboratory index, detailed intervention information and post-intervention follow-up data. Key clinical outcomes were documented.

#### 2.6.1 Pain assessment-11-point numeric rating scale (NRS-11)

The NRS-11 requires the child to directly indicate the intensity of pain using a specific number, where 0 indicates no pain and 10 represents the most severe pain, with intermediate values reflect varying degrees of pain (8).

#### 2.6.2 Blood pressure assessment

During follow-up, blood pressure was measured using a standard cuff sphygmomanometer, with an average of at least three measurements taken on separate days. Clinical success was evaluated after maintaining efficacy for at least 6 months, based on following criteria (3):(1) Cure: No need for antihypertensive medication, and blood pressure remains below the 95th percentile (P95) according to age, gender, and height. (2) Improvement: Blood pressure remains below P95 but still requires antihypertensive medication, or diastolic blood pressure decreases by >15% compared to pre-intervention levels. (3) Ineffective: No change in blood pressure or a decrease that does not meet the above criteria.

#### 2.6.3 Laboratory tests

1. Renin-Angiotensin System Assessment: following diagnostic criteria for renovascular hypertension, selective venous blood sampling was performed during the procedure (prior to embolization therapy) to measure renin levels at three key anatomical sites: 1) the affected renal vein (stenotic side), 2) the contralateral renal vein (healthy kidney side), and 3) the inferior vena cava (serving as systemic circulation control) (9).
2. Renal Function Assessment: kidney function was monitored using Scr, BUN, and 24 h urinary protein excretion (mg/day).

#### 2.6.4 Target organ damage assessment

1. Cardiac Damage: Echocardiography was used to determine the Left Ventricular Mass Index (LVMI). Left ventricular hypertrophy was defined as an LVMI ≥95th percentile for the age and gender of the patient (10).
2. Renal Damage: Evidence of kidney injury was indicated by urinary protein excretion ≥150 mg/d or an elevated Scr level (>upper limit of the age-matched normal range). These criteria were used to assess hypertension-related organ damage.

### 2.7 Statistical analysis

SPSS version 23.0 (IBM Corporation, Armonk, NY, USA) was used for statistical analysis. Measurement data are expressed as mean ± standard deviation and compared using paired *t*-tests or rank-sum tests. A *p*-value <0.05 was considered statistically significant.

## 3 Results

### 3.1 Clinical symptom improvement

We conducted a retrospective analysis of 18 children with renovascular hypertension caused by stenosis in the renal artery branches. Among them, nine patients (50%) presented with headache, while the remaining patients were identified incidentally during routine physical examinations. All patients exhibited severe hypertension, with mean systolic and diastolic blood pressures of 162.8 ± 15.0 mmHg and 114.6 ± 13.5 mmHg, respectively, and required an average of 2.1 ± 1.1 antihypertensive medications. Evaluation of target organ damage revealed hypertensive heart disease (left ventricular hypertrophy) in six (33.3%) and renal impairment in eight patients (44.4%), and the latter uniformly presented elevated 24 h urinary protein excretion (mean: 255.6 ± 232.7 mg/d), including two patients with concomitant elevation in Scr and BUN. Laboratory investigations showed markedly increased pre-intervention peripheral venous plasma renin levels (mean: 100.4 ± 124.4 ng/mL/h), significantly exceeding the normal reference range (0.15–2.33 ng/mL/h), as detailed in Table 1.

**Table 1:** Patient profile.

Characteristic	Value
Men/women (%)	10/8 (55.6/44.4)
Age (y)	9.7 ± 2.3 (range, 6–15)
Clinical symptoms	
Headache (%)	9 (50.0)
NRS-11	6 ± 1.3
Comorbidities	
Hypertensive heart disease	6 (33.3)
Hypertensive renal disease	8 (44.4)
Bp (mmHg)	
SBP	162.8 ± 15.0
DBP	114.6 ± 13.5
No. of antihypertensive drugs	2.1 ± 1.1
Three or more antihypertensive drugs	8 (44.4)
Renal function	
Scr (μmol/L)	50.1 ± 22.1
BUN (mmol/L)	5.4 ± 3.3
24 h urine protein	255.6 ± 232.7
Renin	
Peripheral Venous Blood (Upright position)	100.4 ± 124.4
The affected renal vein (Supine position)	27.0 ± 22.7
The contralateral renal vein (Supine position)	16.8 ± 15.0
The inferior vena cava (IVC) (Supine position)	16.9 ± 14.1

Values are *n* (%) or mean ± SD. NRS-11, Numerical Rating Scale-11. SBP, systolic blood pressure; DBP, diastolic blood pressure. Scr, serum creatinine; BUN, Bblood urea nitrogen. Normal reference values for Scr: 6–12 years: 35–55 μmol/L; 13–18 years: 37–93 μmol/L. Normal reference values for BUN: 2–18 years: 2.7–7.0 mmol/L. Normal renin reference ranges are as follows: 1.31–3.95 ng/mL/h (normal diet, upright position) and 0.15–2.33 ng/mL/h (normal diet, supine position).

**Figure 3:**
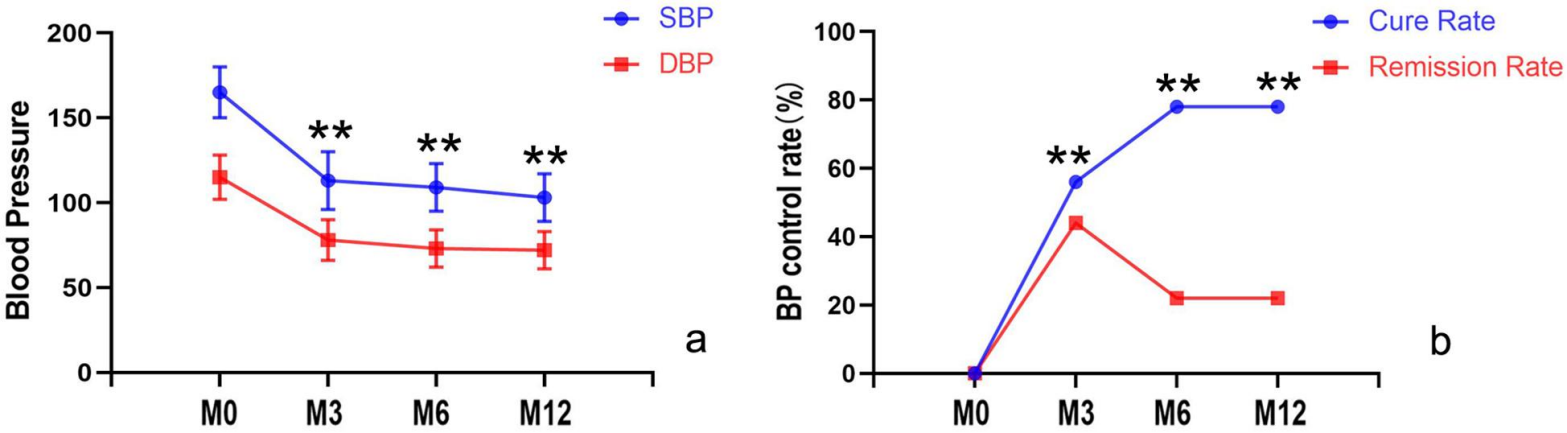
Comparison of systolic and diastolic blood pressure, cure rates, and improvement rates before and after intervention. **(a)** Blood pressure control significantly improved, with both systolic and diastolic pressures markedly reduced compared to pre-intervention levels (*p* < 0.05); **(b)** During follow-up, the cure rate progressively increased (with a corresponding decline in improvement rates). M0, before the intervention; M3, 3 month after intervention; M6, 6 months after intervention; M12, 12 months after intervention.

**Figure 4:**
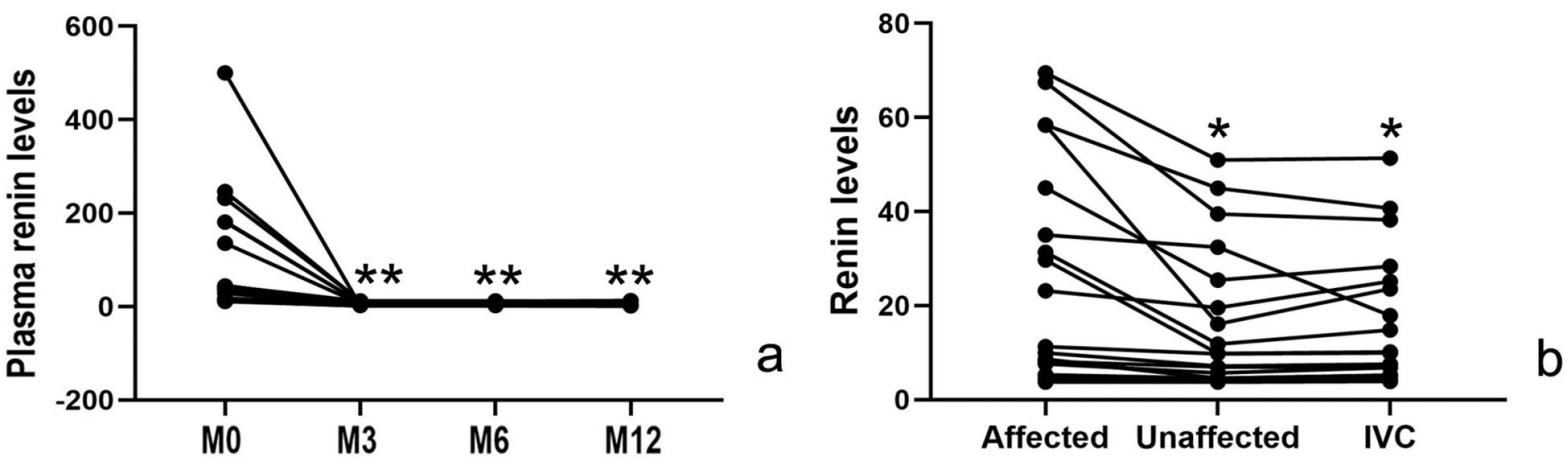
Changes in renin levels: **(a)** post-intervention peripheral venous renin levels were significantly lower than pre-intervention values (*p* < 0.05). **(b)** During the intervention, the affected renal vein demonstrated significantly elevated renin levels compared to both the contralateral renal vein and inferior vena cava (*p* < 0.05). M0, before the intervention; M3, 3 month after intervention; M6, 6 months after intervention; M12, 12 months after intervention.

All patients underwent interventional embolization therapy, and follow-up results showed:

1. Significant clinical improvement, including substantial relief of headache and a marked reduction in NRS-11 scores (*p* < 0.05).
2. Notable improvement in blood pressure control, with both systolic and diastolic pressures showing significant post-intervention reduction (*p* < 0.05) (as shown in Figure 3), accompanied by a decrease in the number of antihypertensive medication requirements (*p* < 0.05).
3. At 3-month follow-up, cure and improvement rates were 55.6% (10/18) and 44.4% (8/18) respectively, at 6–12 months, the cure rate increased to 77.8% (14/18), while the improvement rate showed a corresponding decrease to 22.2% (4/18) as shown in Figure 3.
4. Reversal of target organ damage: At the 3-month echocardiographic follow-up revealed complete resolution of left ventricular hypertrophy in all patients. Both Scr and BUN levels also returned to normal. Additionally, the 24 h urinary protein quantification at 3 months post-intervention was significantly lower than pre-intervention levels. During the 6–12 month follow-up period, except for one child with a mild abnormality, urinary protein indicators of all other children remained at normal levels.
5. Renin dynamics: Intra-intervention measurements showed significantly higher renin levels in stenotic renal veins (27.0 ± 22.7 pg/ml) compared to both the contralateral renal vein(16.8 ± 15.0 pg/ml) and inferior vena cava (16.9 ± 14.1 pg/ml, *p* < 0.05 for both comparisons), with peripheral venous renin levels showing a dramatic post-intervention decline (100.4 ± 124.4 vs. 4.3 ± 2.7 pg/ml at 12 months, *p* < 0.05), as shown in Table 2; Figure 4.

**Table 2:** Clinical variables before and after intervention.

Different time points variable	Base lines	M3		M6		M12	
		Value	*p-*value	Value	*p-*value	Value	*p-*value
NRS-11	2.7 ± 2.9	**0.4** **±** 0.7*	0.007	**0.2** **±** 0.4*	0.007	**0.2** **±** 0.4*	0.007
SBP	162.8 ± 15.0	**113.3** **±** 16.9**	0.000	**109.4** **±** 13.6**	0.000	**103.2** **±** 13.7**	0.000
DBP	114.6 ± 13.5	**77.8** **±** 12.4**	0.000	**73.4** **±** 10.8**	0.000	**71.8** **±** 10.8**	0.000
No. of antihypertensive drugs	2.1 ± 1.1	**1.1** **±** 0.3*	0.003	**0.4** **±** 0.5**	0.000	**0.4** **±** 0.5**	0.000
Cure Rate	/	**10 (55.6)**	/	**14 (77.8)**	/	**14 (77.8)**	/
Remission Rate	/	**8 (44.4)**	/	**4 (22.2)**	/	**4 (22.2)**	/
Scr (μmol/L)	50.1 ± 22.1	46.1 ± 13.9	0.155	**38.1** **±** 10.8*	0.001	**35.6** **±** 7.5*	0.001
BUN(mmol/L)	5.4 ± 3.3	5.1 ± 2.3	0.602	**4.0** **±** 0.7*	0.009	**3.7** **±** 0.7**	0.000
24 h urine protein	255.6 ± 232.7	**129.6** **±** 74.6**	0.000	**95.9** **±** 41.6**	0.000	**85.1** **±** 31.9**	0.000
Renin	100.4 ± 124.4	**5.6** **±** 3.8**	0.000	**5.0** **±** 3.8**	0.000	**4.3** **±** 2.7**	0.000

Values are mean ± SD. Within-group comparisons were performed using paired t-tests or Wilcoxon signed-rank tests.

M3, 3 month after intervention; M6, 6 months after intervention; M12, 12 months after intervention; NRS-11, numerical rating scale-11; SBP, systolic blood pressure; DBP, diastolic blood pressure; Scr, serum creatinine; BUN, blood urea nitrogen.

The asterisks (*/**) on the bold values indicate statistically significant compared to base line, with *p*-values of *p* < 0.05 and *p* < 0.001, respectively.

Normal reference values for Scr: 6–12 years: 35–55 μmol/L; 13–18 years: 37–93 μmol/L. Normal reference values for BUN: 2–18 years: 2.7–7.0 mmol/L. Normal renin reference ranges are as follows: 1.31–3.95 ng/mL/h (normal diet, upright position) and 0.15–2.33 ng/mL/h (normal diet, supine position).

## 4 Discussion

Although the incidence of hypertension in children (1%–5%) is significantly lower than in adults (25%–35%), long-term uncontrolled hypertension can result in serious complications, including hypertensive encephalopathy, stroke, left ventricular hypertrophy, and renal impairment (11–14). Unlike adults, >80% of pediatric hypertension cases have an identifiable underlying cause. Among these renovascular hypertension accounts for 5%–10%.

Hypertension caused by renal artery stenosis (including stenosis of the main trunk or branches) is termed renovascular hypertension, which is often more severe, labile, and more difficult to control than common hypertension. The core pathophysiology involves renal artery stenosis causing inadequate renal blood flow perfusion, which in turn leads to local ischemia. Ischemia stimulates the kidneys to secrete excessive renin, thereby activating the renin-angiotensin-aldosterone system (RAAS), which results in vasoconstriction and sodium-water retention, ultimately leading to elevated blood pressure (15, 16).

In this study, the preoperative plasma renin level was 100.4 ± 124.4 (reference range 1.31–3.95 ng/mL/h), which was significantly higher than the normal range, indicating excessive activation of the RAAS. Moreover, intraoperative renin levels in the renal vein of the affected side were higher than in the contralateral renal vein (27.0 ± 22.7 vs. 16.8 ± 15.0 ng/mL/h, *p* < 0.05; reference range: 0.15–2.33 ng/mL/h). This finding further confirms increased renin secretion from the kidney with renal artery stenosis, reflecting local RAAS overactivation.

Additionally, renal artery stenosis can lead to gradual ischemic atrophy of the kidney and a decline in renal function, a process that typically occurs insidiously and is often difficult to detect. Therefore, for such patients, interventional treatments such as percutaneous angioplasty or renal artery embolization may achieve curative outcomes, significantly improving the prognosis (4, 5).

Preclinical studies have shown that temperature-sensitive embolic agents hold promising potential for vascular embolization therapy. In a study by Zhang et al. (2021), the embolic efficacy of a thermosensitive nanogel (isopropylacrylamide-co-butyl methylacrylate) was evaluated in a rabbit renal artery embolization model. Dynamic perfusion CT imaging revealed that the PIB nanogel rapidly transitioned to a solid state at body temperature, achieving complete occlusion of the renal artery branches. The embolized kidney showed a significant reduction in volume and complete cessation of renal blood flow. No reperfusion or collateral circulation was observed during the 12-week follow-up, confirming durable embolization. Histopathological analysis revealed progressive tissue changes: renal tissue edema with coagulative necrosis at 1 week, structural disintegration by 4 weeks, cellular pyknosis and lysis by 8 weeks (with the gel retained in the vessels), and complete tissue destruction with marked calcification at 12 weeks, demonstrating the long-term efficacy of the PIB nanogel (17).

In another study, Ning et al. (2015) reported the use of a chitosan/β-glycerophosphate (C/GP) thermosensitive hydrogel for embolizing the rete mirabile (REM) in swine. The material exhibited excellent injectability, complete embolization, non-toxicity, and biocompatibility. Post-embolization, the vascular lumen was completely thrombosed, the vessel wall structure remained intact without necrosis or detachment, and no inflammatory response was observed in the surrounding tissues (18). These studies highlight the advantages of thermosensitive embolic agents in vascular occlusion, provide critical evidence for their clinical translation, and suggest their potential value in treating conditions such as renovascular hypertension.

To date, no clinical reports exist on the use of temperature-sensitive embolic agents for the treatment of pediatric renal artery stenosis. However, this study is the first to document the successful percutaneous embolization of renal artery branches using a temperature-sensitive embolic agent in pediatric patients with renovascular hypertension. Following the procedure, all enrolled children exhibited significant clinical improvement, including relief from headaches, reduced NRS-11 pain scores, improved blood pressure control, and decreased reliance on antihypertensive medications. Over time, cure rates increased, with notable mitigation of target organ damage (evidenced by decreased levels of creatinine and blood urea nitrogen) and a marked reduction in renin levels.

These improvements are primarily attributed to the suppression of the RAAS post-intervention, which alleviates excessive contraction of the efferent arteriole, thereby optimizing intraglomerular hemodynamics and facilitating the excretion of metabolic waste products such as creatinine and blood urea nitrogen. Moreover, the reduction in RAAS activity contributes to the normalization of blood pressure, and effective systemic blood pressure control further reduces ongoing high-pressure damage to the kidneys (15, 16). These factors collectively promote the recovery of renal function.

The above results demonstrate that interventional embolization has a significant positive impact on the overall health status of patients, particularly in terms of symptom relief, blood pressure control, and target organ protection. The size of the kidney did not show a significant change post-intervention. As the embolization was performed at the level of tertiary-order vessels, although a certain number of nephrons were lost, it did not substantially alter the overall renal size.

Currently, various materials are available for renal arterial branch embolization in clinical practice. However, thermosensitive embolic agents have distinct advantages in interventional embolization of renal artery branch stenosis. While coils are suitable for embolization of the first- and second-order renal arteries due to their good radiopacity and precise release, they may not completely block blood flow in the distal small branches. This can result in residual stenosis and poor blood pressure control (19). On the other hand, polyvinyl alcohol (PVA) has good biocompatibility and is widely used in clinical practice. However, its inconsistent particle size can lead to aggregation, causing proximal blockage and making it difficult to completely embolize distal micro vessels. This can also affect blood pressure control (20). Similarly, the uniform diameter of acrylic microspheres may ensure vessel-matched occlusion, but it can also lead to incomplete embolization of small branches, inflammatory reactions, and the risk of difficulty in secondary embolization after recurrence (20).

Compared to the limitations of traditional embolic materials—such as coils, PVA particles, and acrylic microspheres—in completely blocking distal micro vessels and their tendency to result in incomplete embolization or recurrence, thermosensitive embolic agents offer unique advantages. The liquid state of thermosensitive embolic agents is not limited by the diameter of the blood vessels and can pass through the stenotic segments without depending on microcatheter superselection. Before turning into a solid state, the agent can permeate into finer vascular branches. Upon reaching body temperature, the material rapidly undergoes a phase change to form a solid polymer, thereby achieving complete occlusion of the vascular system.

Although thermosensitive embolic agents are in a liquid form, they show excellent targeting and safety in renal artery branch embolization. These characteristics are mainly attributed to the unique vascular anatomy and hemodynamic features of the kidneys. According to the classical theory of renal segment division, the kidneys can be divided into five relatively independent perfusion segments: apical, upper, middle, lower, and posterior, each of which is supplied by the corresponding segmental artery (21). Because no effective collateral circulation exists between the renal artery branches (i.e., functional terminal arteries), the blood flow between the segments is relatively independent. This anatomical feature provides an ideal biological basis for the clinical use of thermosensitive embolic agents. When using thermosensitive embolic agents and other liquid embolic materials for superselective embolization, the embolic agent can accurately act on the target vessel and does not cause nontarget embolism due to reflux or collateral compensation. This significantly reduces the risk of nontarget embolization, prevents ischemic damage to normal renal tissue, and improves the overall safety and effectiveness of treatment, ultimately leading to long-term stable blood pressure control.

After interventional embolization, stenosis and abnormal hemodynamics of the target vessels are corrected, renal artery blood flow distribution is improved, and excessive activation of the RAAS is effectively inhibited, eventually leading to significant blood pressure control. This may explain why satisfactory blood pressure control and normalization of renin levels are achieved after intervention.

Ethanol, a potent vascular sclerosing agent, can rapidly induce thrombotic occlusion. However, its invisibility, diffusibility, and high reflux risk may lead to misembolization, renal parenchymal injury, and systemic complications—such as hemolysis and heart failure with significant procedural risks (22). In contrast, the radiopacity and visibility of thermosensitive embolic agents (iodixanol) can meet intraoperative tracking needs, and embolization can be stopped when a columnar shape appears in the blood vessels. During the procedure, the injection speed is kept very low, and even if reflux occurs, the injection can be stopped in time to reduce the occurrence of reflux and ectopic embolization. In our study, no adverse events were reported, in contrast to the complications reported with ethanol—such as renal dysfunction.

Several shortcomings that still exist in the use of thermosensitive embolic agents: (1) Stringent phase-change control is required: Thermosensitive embolic agents need to be precisely used near the phase-change temperature, which is typically around 30–35℃. During the procedure, the catheter must be continuously cooled, for instance by flushing it with cold heparin saline during injection, to avoid premature phase-change-induced catheter blockage. If the temperature is not properly controlled, solidification can occur inside the catheter. (2) The procedure is complicated: Combining imaging guidance, such as CT perfusion or digital subtraction angiography (DSA), is necessary to monitor the embolization effect in real time. The procedure demands a high level of technical expertise, because the injection speed needs to be strictly maintained within the 1.5–3 ml/min range. Deviations can increase the risk of ectopic embolization or catheter occlusion. (3) Irreversible embolization range: Although thermosensitive embolic agents can reduce the risk of reflux and ectopic embolization, they cannot ensure that these will not occur. If excessive embolization or incorrect embolization occurs, it cannot be reversed through temperature regulation, potentially leading to irreversible tissue damage, such as local infarction or calcification after renal artery embolization. (4) Limitations of preclinical studies: Most current research is based on animal models, such as rabbit or pigs. Clinical experience is still limited, and long-term safety and efficacy of these agents require further investigation through extended follow-up (17, 18). (5) Insufficient long-term safety data: Although animal experiments have shown no recanalization after 12 weeks, the long-term biocompatibility including risk of chronic inflammation or foreign body reactions has yet to be fully validated (17).

This study is the first to report the clinical application of thermosensitive embolic agents in the treatment of renovascular hypertension caused by renal artery branch stenosis in pediatric patients. Thermosensitive embolic agents have shown unique advantages in vascular embolization. In their liquid state, these agents can penetrate even the smallest distal branches, and upon reaching body temperature, they rapidly transition to a solid state, achieving complete and targeted vascular occlusion. This characteristic overcomes the limitations of conventional embolic materials, such as coils, PVA particles, and acrylic microspheres, which often fail to fully obstruct the distal microvasculature. Hence, thermosensitive agents significantly enhance both the thoroughness and the precision of embolization procedures. The clinical outcomes observed were significant, with notable postoperative improvements in blood pressure control, normalization of renin levels, and mitigation of target organ damage.

However, limitations remain, including the need for strict temperature control requirements for phase transition, procedural complexity, the irreversible nature of embolization, and insufficient long-term safety data due to the predominance of preclinical animal studies. Further optimization of the operational protocols and additional clinical experience are necessary to validate the long-term efficacy and safety of this treatment.

## Data Availability

The original contributions presented in the study are included in the article/Supplementary Material, further inquiries can be directed to the corresponding author.
